# Research Priorities for Zoonotic and Pandemic Influenza Vaccines: Evidence and Recommendations from the WHO Public Health Research Agenda for Influenza (2024 Update)

**DOI:** 10.3390/vaccines13121206

**Published:** 2025-11-29

**Authors:** Wenqing Zhang, Benjamin J. Cowling, John S. L. Tam, Thomas Abraham, Hualan Chen, Keenan Duggal, Wei Xin Khong, Sebastian Maurer-Stroh, Arnold S. Monto, Sergejs Nikisins, Tulio de Oliveira, Yuelong Shu, Cecile Viboud, Richard Webby, Sylvie van der Werf, Jessica Wong, Jean-Michel Heraud

**Affiliations:** 1 Global Influenza Programme, World Health Organization, CH-1211 Geneva, Switzerland; zhangw@who.int (W.Z.); nikisinss@who.int (S.N.); 2 School of Public Health, The University of Hong Kong, Hong Kong SAR 999077, China; bcowling@hku.hk (B.J.C.); wongytj@hku.hk (J.W.); 3 Department of Applied Biology and Chemical Technology, The Hong Kong Polytechnic University, Hong Kong SAR 999077, China; john.sl.tam@connect.polyu.hk; 4 Journalism and Media Studies Centre, The University of Honk Kong, Hong Kong SAR 999077, China; abrahk@gmail.com; 5 China Harbin Veterinary Research Institute, Harbin 150001, China; chenhualan@caas.cn; 6 New York University Grossman School of Medicine, New York, NY 10016, USA; keenanduggal@gmail.com; 7 Communicable Diseases Agency, Singapore 307684, Singapore; khong_wei_xin@cda.gov.sg; 8 GISAID Data Science Centre @ A*STAR Bioinformatics Institute (BII), Singapore 138671, Singapore; sebastianms@gisaid.org; 9 Yong Loo Lin School of Medicine, National University of Singapore, Singapore 117558, Singapore; 10 Department of Biological Sciences, National University of Singapore, Singapore 117558, Singapore; 11 School of Public Health, University of Michigan, Ann Arbor, MI 48109, USA; asmonto@umich.edu; 12 Centre for Epidemic Response and Innovation, Stellenbosch University, Stellenbosch 7602, South Africa; tulio@sun.ac.za; 13 School of Public Health, Shenzhen Campus of Sun Yat-sen University, Shenzhen 518107, China; shuylong@mail.sysu.edu.cn; 14 Fogarty International Center, National Institutes of Health, Bethesda, MD 20892, USA; viboudc@mail.nih.gov; 15 St. Jude Children’s Research Hospital, Memphis, TN 38105, USA; richard.webby@stjude.org; 16 Molecular Genetics of RNA Viruses Unit, Institut Pasteur, 75015 Paris, France; sylvie.van-der-werf@pasteur.fr; 17 Virology Unit, Institut Pasteur de Madagascar, Antananarivo 101, Madagascar; 18 Department of Global Health, Institut Pasteur, 75015 Paris, France

**Keywords:** zoonotic influenza, pandemic preparedness, vaccines, One Health, vaccine uptake, predictive modelling, RCCE

## Abstract

Zoonotic influenza viruses, including highly pathogenic avian influenza and swine-origin variants, continue to cause sporadic human infections with, in some cases, high case fatality rates and potential for sustained human-to-human transmission. The COVID-19 pandemic underscored both the possibilities of rapid vaccine innovation and the persistent challenges in equitable access and public trust. This paper synthesizes the vaccine-related priorities from the 2024 update of the World Health Organization Public Health Research Agenda for Influenza, integrating evidence from systematic literature reviews commissioned, expert consultations, and analysis of lessons learned from recent health emergencies, to outline a research and policy roadmap for zoonotic and pandemic influenza vaccine preparedness. Key research priorities identified include development of broadly protective animal and human vaccines; improved understanding of correlates of protection; rapid and scalable manufacturing platforms; predictive modelling for strain selection; and targeted communication strategies to strengthen uptake. Experts have considered that implementing these priorities will require One Health integration, sustained investment, harmonized regulatory frameworks, and proactive community engagement to ensure that advances in vaccine science translate into timely, equitable public health protection.

## 1. Introduction

Influenza viruses remain a major cause of morbidity and mortality worldwide, with seasonal epidemics responsible for an estimated 300,000–500,000 respiratory deaths annually [1]. In addition to seasonal strains, zoonotic influenza viruses such as H5N1, H5N6, H7N9, and variant swine-origin viruses continue to cause sporadic human infections, sometimes associated with high case fatality rates and severe clinical outcomes [2]. The risk of a novel, efficiently transmissible influenza virus emerging from animal reservoirs remains a constant global threat.

Over the past two decades, the frequency of zoonotic influenza spillovers has increased, driven by agricultural intensification, wildlife trade, and land-use changes that heighten animal–human contact [3]. Additional drivers include the unprecedented scale of international travel, which accelerates the global dissemination of emerging viruses, and rising population density in rapidly urbanising settings, which increases contact rates and can amplify early transmission. Both factors contribute to the growing likelihood that zoonotic spillovers can seed larger outbreaks.

Strengthening surveillance at the human–animal interface through a One Health approach is therefore critical to ensure the timely detection and characterization of viruses with pandemic potential and to inform vaccine design. The World Health Organization (WHO) global influenza programme has developed a Tool for Influenza Pandemic Risk Assessment (TIPRA) highlighting how animal and human data can jointly guide vaccine research priorities [4].

Equally important are the social and behavioural determinants of vaccine acceptance [5], which have emerged as key barriers to achieving high coverage even where supply exists.

The COVID-19 pandemic demonstrated the potential for rapid vaccine development, with novel platforms such as mRNA and viral vectors enabling the delivery of effective products within months. However, it also revealed gaps in manufacturing agility, equitable access, regulatory coordination, and public confidence [6,7]. These lessons are highly relevant to pandemic influenza preparedness, where the window for intervention is often short and the scale of deployment unprecedented.

**Figure 1:**
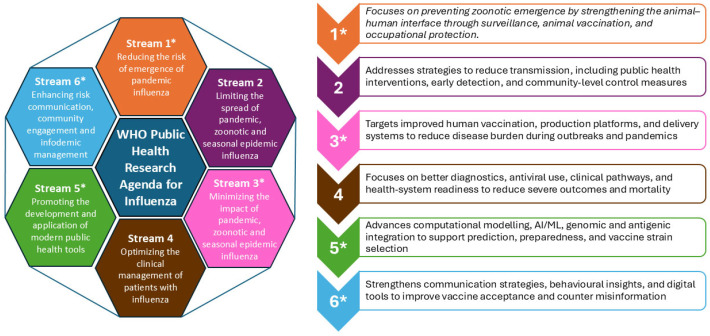
The six research streams of the WHO Public Health Research Agenda for Influenza (2024 update). The 2024 update of the WHO Public Health Research Agenda for Influenza organizes research needs into six thematic [8] streams spanning the animal–human interface, transmission dynamics, human vaccination, clinical management, modern public health tools, and risk communication. Streams marked with an asterisk correspond to those that include explicit vaccine-related research priorities for zoonotic and pandemic influenza.

The WHO Public Health Research Agenda for Influenza, first published in 2009 and updated in 2017, was revised in 2024 following systematic literature reviews, expert consultations, and an assessment of lessons learned from recent health emergencies [8]. The 2024 update has organized research needs into six thematic streams (Figure 1). Vaccine-related priorities are distributed across four of these steams (1, 3, 5 and 6), which together provide direction for advancing research on zoonotic and pandemic vaccine.

Although the updated research agenda comprises six streams, this Perspective focuses on the four streams that include explicit research priorities on zoonotic or pandemic influenza vaccine. Stream 2 (“Limiting the spread of pandemic, zoonotic and seasonal epidemic influenza”) and Stream 4 (“Optimizing the clinical management of patients with influenza”) do refer to the role of vaccines, such as their impact on transmission dynamics or clinical outcomes, but they do not define vaccine-specific research priorities for zoonotic or pandemic influenza. Nevertheless, both streams remain essential pillars of pandemic preparedness, complementing vaccine-focused work through strengthened non-pharmaceutical interventions, enhanced surveillance, health-system readiness, and improved clinical management.

This article synthesizes the vaccine-related priorities identified in the updated agenda, contextualized with scientific and operational evidence from literature reviews and meetings conducted with experts during the process of updating the research agenda. The research agenda aims to provide a consolidated strategic roadmap for zoonotic and pandemic influenza vaccine research for the next decade, combining scientific innovation with policy and implementation readiness.

## 2. Current Landscape of Influenza Vaccines

### 2.1. Human Seasonal and Pandemic Vaccines

Most licensed human influenza vaccines are egg-based, inactivated split-virion formulations. These vaccines are safe and widely used but have limitations in breadth of protection, durability of immunity, and production speed [9]. Antigenic mismatch, particularly for rapidly evolving subtypes, can markedly reduce effectiveness. Advances in cell-based, recombinant protein, and mRNA platforms offer improved antigenic fidelity and manufacturing agility, but their integration into routine vaccination programmes and pandemic readiness planning remains incomplete [10,11].

Pandemic influenza vaccines currently rely on a network of National Influenza Centres, WHO Collaborating Centres and Essential Regulatory Laboratories (known as GISRS—Global Influenza Surveillance and Response Systems) to develop and distribute candidate vaccine viruses [12]. Stockpiling of antigens and adjuvants for priority pandemic subtypes (e.g., H5, H7) is ongoing, but production timelines, manufacturing surge capacity, and equitable access remain significant constraints [13,14].

Despite technological progress such as cell-based and recombinant platforms, global seasonal influenza vaccine coverage remains low in low- and middle-income countries [5] due to limited production capacity, cold-chain challenges, and unpredictable financing [15]. Initiatives such as the WHO mRNA Technology Transfer Programme are exploring regional manufacturing hubs to close this gap [16].

### 2.2. Animal Vaccines

Vaccination of poultry, swine, and other at-risk species can reduce viral load and shedding, thereby lowering the risk of zoonotic transmission [17]. With 78–97% efficacy, current avian influenza vaccines offer a powerful tool to prevent poultry culling, safeguard livelihoods, and strengthen food security [18]. However, uptake varies across regions due to economic, logistical, and policy barriers commonly found for many animal vaccines [19]. Antigenic mismatch is a recurring problem where vaccine strain updates lag behind field virus evolution.

Recent field evidence from France demonstrates that large-scale duck vaccination against H5N1 HPAI significantly reduced viral circulation and culling needs, validating the potential of vaccination to curb zoonotic risk [20]. However, uneven policy frameworks and limited data on field efficacy hinder harmonized adoption. Comparative studies between vaccinated and unvaccinated flocks could better quantify the public-health benefit of animal immunization in reducing spillover risk [18].

### 2.3. Lessons from COVID-19 Vaccine Development

The COVID-19 response showed that with coordinated investment and regulatory flexibility, vaccine development timelines can be compressed from years to months. This global response also revealed important trade-offs. The unprecedented financial mobilisation redirected resources away from other priority programmes in many countries, and global supply chains experienced substantial shortages of vials, bioreactor bags, adjuvants, and fill-and-finish capacity [21]. Human expertise and laboratory infrastructure were often diverted to COVID-19 projects, slowing progress in other areas of infectious disease prevention and control (e.g., research activities, diagnostic capacities, treatment programmes and routine immunization services) [22]. This perceived success of ‘ultra-rapid’ COVID-19 vaccine development may create an unrealistic expectation that similar timelines will be easily achievable for future pandemics without comparable investment, risk-sharing mechanisms, or global coordination.

Nevertheless, pandemic influenza preparedness can benefit from platform technologies, decentralized manufacturing, real-time genomic surveillance, predictive modelling, artificial intelligence and machine learning (AI/ML), and transparent communication strategies that address misinformation.

## 3. Research Priorities for Zoonotic and Pandemic Influenza Vaccines

Vaccine related priorities in the 2024 WHO Public Health Research Agenda for Influenza are summarized in Table 1, which organizes key research directions by thematic stream.

### 3.1. Animal–Human Interface (Highlights from Stream 1)

The 2024 WHO research agenda for influenza highlights vaccination as a dual strategy to reduce zoonotic risk. Indeed, animal vaccination lowers viral replication and transmission in reservoir species such as poultry and swine, while human vaccination in occupationally exposed groups (e.g., poultry workers, veterinarians, and market handlers) provides an added layer of protection.

Research priorities include the development of cost-effective, broadly protective animal vaccines, such as multivalent or multi-pathogen formulations that can be integrated into existing animal health programs [19,23] and mechanisms to rapidly update vaccine strains based on surveillance data. Innovative approaches, including recombinant live vaccines and scalable delivery systems, are also needed to enable mass immunization in large-scale farming or high-risk wildlife populations.

Further research should quantify the ecological and epidemiological benefits of combined animal–human vaccination strategies in mixed-species production systems, which may concurrently reduce outbreak magnitude and occupational exposure.

### 3.2. Human Pandemic Vaccine Innovation (Highlights from Stream 3)

A central research direction is the pursuit of universal or broadly protective human influenza vaccines capable of inducing immunity against conserved viral epitopes, thereby offering cross-protection against drifted or novel strains. Building on lessons from COVID-19, novel platforms such as mRNA, viral vectors, and nanoparticle-based vaccines should be advanced for rapid adaptability to emerging viruses with pandemic potential. It is important to note that rapid adaptability of platforms should not be interpreted as a substitute for the development of vaccines with broader or universal protection. Adaptability accelerates the response to emerging strains, whereas breadth aims to reduce dependence on frequent updates. Both approaches are complementary and must be pursued in parallel to strengthen pandemic readiness.

Defining robust correlates of protection beyond neutralizing antibody titres, including mucosal and T-cell mediated responses, is considered essential to guide vaccine design and dosing strategies.

The research agenda also calls for the optimization of dose-sparing strategies, such as the use of potent adjuvants and alternative administration routes, to maximize coverage in the event of limited supply. Regulatory science should support adaptive trial designs and leverage real-world effectiveness data to accelerate licensure. Thermostable, single-dose vaccines are highlighted as a research priority for use in resource-limited settings.

Improvements in manufacturing agility are critical, with a goal of reducing the interval from strain identification to vaccine availability. Complementary initiatives such as CEPI’s 100-Days Mission outline practical pathways to reduce vaccine development timelines from strain identification to large-scale rollout [24]. Comparative immunogenicity studies across mRNA, viral-vector, and nanoparticle platforms can elucidate conserved immune correlates for broadly protective vaccines.

### 3.3. Enabling Data and Predictive Tools (Highlights from Stream 5)

To anticipate antigenic changes before they compromise vaccine effectiveness, the agenda stresses the integrated use of genomic, antigenic, and epidemiological data. Predictive modelling approaches, particularly those employing machine learning algorithms such as EVEscape and PandoGen, are seen as promising for identifying mutations linked to immune escape or altered transmissibility [25,26]. Such tools depend on global data standards and interoperable governance frameworks that allow near real-time sharing and analysis of surveillance outputs, as crucially provided through the GISAID data science platform [27]. In the context of pandemic preparedness, these predictive capacities can enable earlier initiation of candidate vaccine virus development and manufacturing. Integration of antigenic cartography and real-time genomic data from GISAID has revolutionized vaccine strain selection by allowing near-real-time tracking of viral evolution through GISAID and NextStrain [27,28]. Continued investment in AI/ML–based forecasting tools will further enhance the predictive capacity of global influenza surveillance.

### 3.4. Communication and Strategies for Uptake Improvement (Highlights from Stream 6)

Vaccine effectiveness is contingent on public acceptance, making risk communication and community engagement (RCCE) an integral component of preparedness. The research agenda calls for the design of tailored communication strategies that address local sociocultural contexts, linguistic diversity, and trust dynamics.

Digital epidemiology tools, such as AI-driven infodemic monitoring systems like VaccineLies and CoVaxLies, offer real-time insight into misinformation trends, enabling proactive countermeasures [29,30]. The engagement of high-exposure occupational groups as trusted messengers is recommended to improve uptake, particularly in rural and high-risk settings. All messaging should align with WHO’s six communication principles, ensuring information is Accessible, Actionable, Credible, Relevant, Timely, and Understandable, to strengthen public trust in vaccination programmes [31].

Beyond monitoring misinformation, participatory communication models that involve local leaders, healthcare workers, and veterinarians have shown measurable improvements in vaccine uptake and trust [32]. Evidence-based behavioural interventions can complement these approaches to counter misinformation [33].

## 4. Cross-Cutting Enablers

Key enablers include operationalizing One Health integration for joint surveillance and vaccine decision-making, harmonizing regulatory processes for rapid approval, expanding manufacturing surge capacity through regional hubs, and embedding equity into allocation frameworks to avoid the disparities seen during COVID-19.

Regional manufacturing networks, such as those established under the WHO mRNA technology transfer hub in Africa and South-East Asia, illustrate how distributed production can strengthen supply resilience during a pandemic [16]. In parallel, convergence among national regulatory authorities, fostered through the International Coalition of Medicines Regulatory Authorities (ICMRA), can streamline emergency authorizations while maintaining safety standards [34].

While regional manufacturing hubs are essential to reduce dependency on a small number of global producers, they must be complemented by robust and equitable allocation mechanisms. Evidence from the COVID-19 response shows that increased production capacity alone is insufficient to ensure timely access in all regions, particularly without predefined pathways for redistribution. Complementary frameworks, including pre-negotiated donation agreements (as mentioned in article 14.2 of the WHO Pandemic Agreement) [35], pooled procurement instruments that allow countries to collectively purchase vaccines through advance arrangements, and global governance structures such as the WHO Pandemic Influenza Preparedness (PIP) Framework, which embeds mandatory benefit-sharing obligations through its Standard Material Transfer Agreements (SMTA2) [36], are essential to help mitigate the inequities in vaccine access observed during the COVID-19 pandemic. Pandemic preparedness efforts could benefit from additional operational guidance on when and how donation and redistribution mechanisms might be activated.

## 5. Policy Recommendations and Strategic Roadmap

Governments should consider institutionalizing pandemic influenza vaccine R&D within national health security strategies, with sustained funding in inter-pandemic periods. Coordinated efforts by international agencies can enhance cross-sectoral collaboration, standardize technical processes, and foster regional consortia for vaccine development. A WHO guidance document has been recently published to assist countries to update their national deployment and vaccination plans for vaccines against pandemic influenza [37]. New data technology platforms including genomic and epidemiological inputs should be developed with robust governance. Community engagement should be a continuous process, with investment in RCCE capacity in high-risk settings.

Global preparedness financing instruments, including the World Bank’s pandemic fund and CEPI’s long-term R&D mechanisms, should be institutionalized to sustain vaccine research and innovation between crises. Embedding social-science and implementation research within vaccine programmes will help ensure that scientific advances translate into equitable access and community acceptance. At the same time, the forthcoming WHO pandemic agreement seeks to strengthen global governance for pandemic prevention, preparedness and response, promoting coordination, solidarity and equitable access to vaccines and other health countermeasures.

## 6. Conclusions

The 2024 update of the WHO Public Health Research Agenda for Influenza provides a comprehensive and feasible framework for advancing zoonotic and pandemic influenza vaccine research over the next decade. Progress requires simultaneous advances in scientific innovation, operational readiness, and public trust. Sustained investment, multidisciplinary collaboration, and equity-focused strategies are essential to ensure that when the next influenza pandemic emerges, vaccines are available, accessible, and acceptable in time to mitigate morbidity, mortality, and social disruption.

## Figures and Tables

**Table 1:** Summary of vaccine-related research priorities for zoonotic and pandemic influenza, organized by research agenda stream.

Stream	Vaccine Related Research Priorities
Stream 1: Reducing the risk of emergence of pandemic influenza	Broadly protective and cost-effective animal vaccines Occupational human vaccination strategies
Stream 3: Minimizing the impact of pandemic, zoonotic and seasonal epidemic	Universal or broadly protective human vaccines Novel platforms (e.g., mRNA, viral vector, nanoparticles) Correlates of protection (in particular for new vaccines) Dose-sparing strategies Thermostable formulations Manufacturing agility
Stream 5: Promoting the development and application of modern public health tools	Integration of genomic, antigenic, and epidemiological data Predictive modelling for strain selection AI/ML-based early antigenic drift forecasting
Stream 6: Enhancing risk communication, community engagement (RCCE) and infodemic management	Tailored RCCE strategies Digital/AI-based infodemic monitoring Engagement of high-risk occupational groups

## Data Availability

All the data are present in the manuscript.
